# Hyperspectral imaging combined with GA‐SVM for maize variety identification

**DOI:** 10.1002/fsn3.3984

**Published:** 2024-04-03

**Authors:** Fu Zhang, Mengyao Wang, Fangyuan Zhang, Ying Xiong, Xinyue Wang, Shaukat Ali, Yakun Zhang, Sanling Fu

**Affiliations:** ^1^ College of Agricultural Equipment Engineering Henan University of Science and Technology Luoyang China; ^2^ Collaborative Innovation Center of Advanced Manufacturing for Machinery and Equipment of Henan Province Luoyang China; ^3^ College of Agriculture/Peon Henan University of Science and Technology Luoyang China; ^4^ Wah Engineering College, University of Wah Wah Cantt Pakistan; ^5^ School of Physical Engineering Henan University of Science and Technology Luoyang China

**Keywords:** genetic algorithm, hyperspectral imaging technology, maize, support vector machine, variety identification

## Abstract

The demand for identification of maize varieties has increased dramatically due to the phenomenon of mixed seeds and inferior varieties pretending to be high‐quality varieties continuing to occur. It is urgent to solve the problem of efficient and accurate identification of maize varieties. A hyperspectral image acquisition system was used to acquire images of maize seeds. Regions of interest (ROI) with an embryo size of 10 × 10 pixel were extracted, and the average spectral information in the range of 949.43–1709.49 nm was intercepted for the subsequent study in order to eliminate random noise at both ends. Savitzky–Golay (SG) smoothing algorithm and multiple scattering correction (MSC) were used to pretreat the full‐band spectrum. The feature wavelengths were screened by successive projection algorithms (SPA), competitive adaptive reweighted sampling (CARS) single screening, and two combinations of CARS‐SPA and CARS + SPA, respectively. Support vector machines (SVMs) and models optimized based on genetic algorithm (GA), particle swarm optimization (PSO) were established by using full bands (FB) and feature bands as the model input. The results showed that the MSC‐(CARS‐SPA)‐GA‐SVM model had the best performance with 93.00% of the test set accuracy, 8 feature variables, and a running time of 24.45 s. MSC pretreatment can effectively eliminate the scattering effect of spectral data, and the feature wavelengths extracted by CARS‐SPA can represent all wavelength information. The study proved that hyperspectral imaging combined with GA‐SVM can realize the identification of maize varieties, which provided a theoretical basis for maize variety classification and authenticity identification.

## Graphical abstract

An innovative method of genetic algorithm (GA) and particle swarm optimization (PSO) based on hyperspectral imaging technology to optimize the traditional support vector machine (SVM) model was proposed to achieve non‐destructive, efficient, and accurate classification of maize varieties. The results showed that the MSC‐(CARS‐SPA)‐GA‐SVM model had the best performance with 93.00% of the test set accuracy, 8 feature variables, and a running time of 24.45 s, which provided a theoretical basis for maize variety classification and authenticity identification.

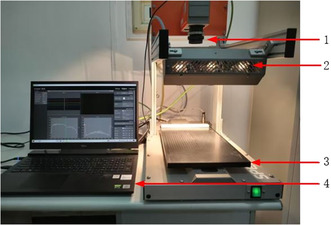

## 1 INTRODUCTION

As a large population country, grain plays an irreplaceable role in China. Grain production and quality will directly affect people's lives, international economic construction, and social stability. The large amount of nutrients contained in maize makes it not only a source of starch and important grain crops but also widely used to make animal feed (Li, 2020). As the main food crop for human beings, people's demand for maize has changed from quantity to quality. Due to the frequent occurrence of mixed seeds between high‐quality and poor‐quality maize, which has caused huge economic losses, the demand for the identification of maize varieties has increased dramatically (Huang, Liu, et al., 2022; Huang, Zhou, et al., 2022). It is necessary to solve the problem of how to identify maize varieties efficiently and accurately, which is of great significance to ensuring food security.

Commonly used variety identification mainly includes near‐infrared spectroscopy, chemical composition analysis, manual identification, and computer vision (Huang, Liu, et al., 2022; Huang, Zhou, et al., 2022; Jiao et al., 2021; Xin & Yang, 2020; Zhang, Li, et al., 2022; Zhang, Zhang, et al., 2022), which mostly have the disadvantages of long cycle time, complicated operation, and high cost. Hyperspectral imaging techniques, which integrate spectral and image information, have been widely used in variety identification studies of rice, soybean, and wheat (Wu et al., 2021). An SVM parameter optimization model based on an improved genetic algorithm (IGA) enhanced the classification effect of Gannan navel oranges with an accuracy of 98.00% (Huang et al., 2023). Based on deep learning combined with hyperspectral imaging technology for the identification of *Fritillaria thunbergerii* varieties, the CNN model had the highest recognition accuracy (Kabir et al., 2022). The CARS‐SVM model had the best classification results, with 100% and 85% in the training and prediction sets based on hyperspectral image technology to identify six varieties of Chinese wolfberry (Tang et al., 2021). LW‐NIR hyperspectral reflectance imaging coupled with a two‐band ratio and an improved watershed segmentation algorithm was used to realize the detection of early decay in citrus. The image‐level detection method proposed in this study obtained a total success rate of 92% for all fruit (Tian et al., 2021). Hyperspectral reflectance imaging coupled with optimal wavelength selection and an improved watershed segmentation algorithm was used to detect early bruises on apples, which obtained recognition rates of 93.3%, 92.2%, and 92.5% for healthy, bruised, and overall apples, respectively (Tian et al., 2023). Hyperspectral imaging was combined with three discriminant models to identify four barley seed varieties. The recognition rates of the SPA‐K‐nearest neighbor model were 93.71% (Sun et al., 2021). The deep convolutional neural network VGG16 was improved by combining deep learning and machine vision, and the classification accuracy was up to 96.70% (Yang et al., 2021). The accuracy of the training and test sets of the (SG‐FD)‐CARS‐SVM model for sweet corn variety identification based on hyperspectral imaging was 94.07% and 94.86%, respectively (Zhou et al., 2020). The classification accuracy of the MSC‐CARS‐BAS‐WOA‐SVR model was the highest based on near‐infrared spectroscopy (Xu et al., 2023). Nondestructive detection of external defects of Kubota based on hyperspectral detection technology, the classification reached an accuracy of 96.77% for the prediction set of the CARS‐GS‐SVM model (Zhang et al., 2023). Lodging resistance of maize varieties was identified by using hyperspectral imaging technology combined with machine learning algorithms; the SPA‐SVM model worked best with 97.40% and 98.33% in the training and test sets, respectively (Zhang, Li, et al., 2022; Zhang, Zhang, et al., 2022). Non‐destructive identification of black wolfberry and prickly berry based on hyperspectral imaging technology, the recognition rates of ELM and SVM models established by FS and SPA are 100% (Zhao et al., 2021). The accuracy of the CSA‐SVM model under the combination of spectral and texture features reached 96.57% based on hyperspectral imaging technology (Wang et al., 2021). The SVM model was the best for grape variety identification using hyperspectral techniques, with classification accuracy of 93.06% and 90.01% for the training and test sets of red grapes, respectively (Cheng, 2020). There are some problems with model complexity, low model stability, and low test set accuracy in the studies, and the SVM optimization model is rarely used in the identification of maize varieties.

At present, scholars have conducted some research on crop variety identification and related quality index detection. However, current research on the identification of maize varieties is still in the preliminary exploration stage. SVM was used mostly or optimized by single algorithm, which had low classification accuracy and was greatly affected by parameters. Characteristic wavelengths are mostly selected by CARS and SPA, which is easy to cause some problems, such as part of the effective wavelength information missing, classification model prediction accuracy, and operation efficiency degradation. Therefore, GA and PSO were used for modeling analysis in this paper. Characteristic wavelengths were extracted by CARS and SPA single extraction and combined screening methods of CARS‐SPA and CARS + SPA after SG and MSC pretreatment. Hyperspectral detection combined with various optimization models was used to detect the adulteration of maize varieties, and classification models with short running times and high accuracy were selected.

## 2 MATERIALS AND METHODS

### 2.1 Materials

The maize seed samples used in the experiment were all from the College of Agriculture, Henan University of Science and Technology, Luoyang city, Henan Province, P. R. China. Five varieties of maize seeds with normal quality and flawless appearance were manually selected and labeled as varieties from 1 to 5, respectively. Sixty seeds were selected for each variety, and a total of 300 test samples were collected.

### 2.2 Test instruments and equipment

**FIGURE 1:**
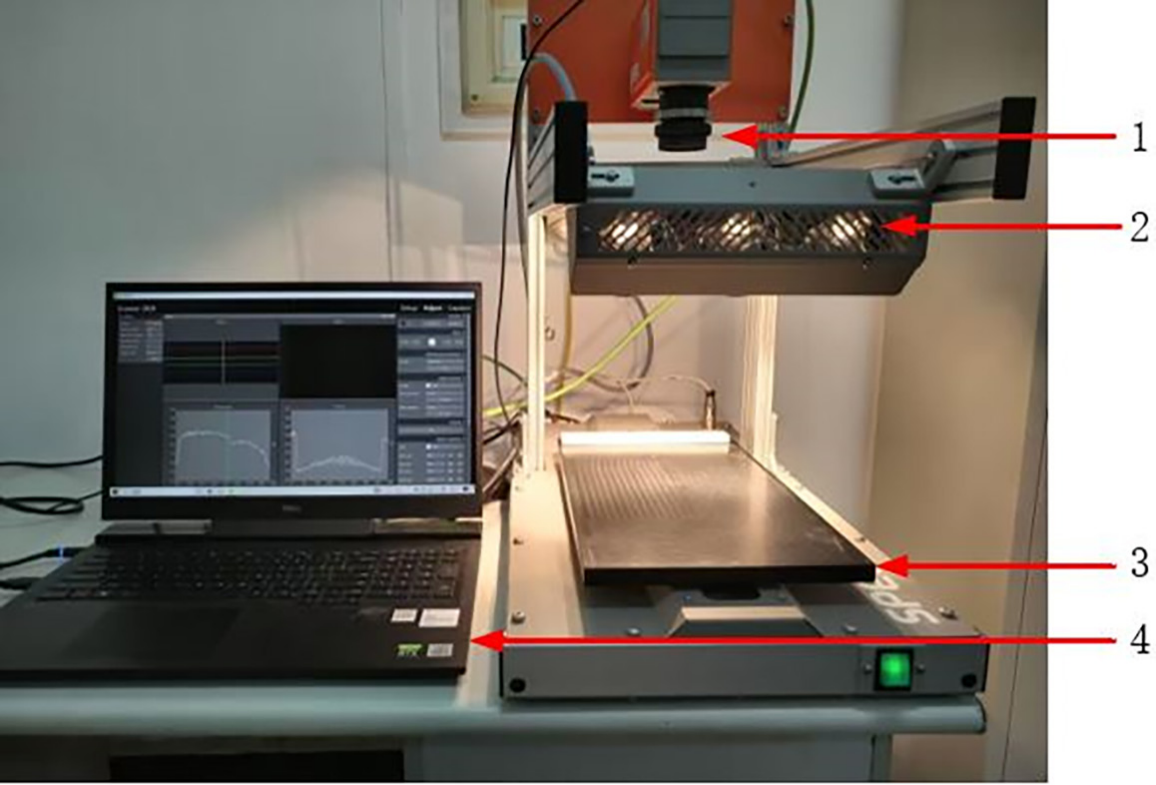
Hyperspectral imaging system. (1) Hyperspectral imager (SPECIM FX17e, Finland); (2) Halogen lamp light source; (3) Electronically controlled displacement table; (4) Computer.

The hyperspectral imaging acquisition equipment used in this experiment mainly included a hyperspectral imager (SPECIM FX17e, Finland), a halogen lamp light source, an electronically controlled displacement table, a dark box, and a computer, as shown in Figure 1. The scanning range of the hyperspectral imager was 900–1700 nm, spectral resolution was 8 nm, and the total number of spectral bands was 224. Lumo Scanner software was used to collect and save the hyperspectral image information; ENVI 5.3 software developed by Harris Geospatial Solutions in the United States was used to extract the spectral data of the samples; the Unscrambler X10.4 software developed by CAMO Software in Norway was used for spectral pretreatment; and MATLAB 2016b software developed by MathWorks in the United States was used to extract the characteristic wavelengths and modeling analysis.

### 2.3 Spectral acquisition method

The instrument needed to be preheated for 30 min before the test in order to ensure the light source output stable light intensity. The best parameters were determined after several tests and debugging to make the collected images clear and undistorted: the moving speed of the displacement platform was 18.28 mm/s, the exposure time was 6.5 ms, the distance between the sample and the hyperspectral camera lens was 320 mm, and the data acquisition frequency was 50 Hz.

Image acquisition was carried out in a dark box to avoid the influence of external light sources during the test process. Seeds were pasted on sticky black tool paper with proper spacing to prevent the maize seeds from rolling on the electronically controlled mobile carrier table. The images of one variety, 60 grains of maize, were collected each time. In order to reduce the influence of dark current, noise, and the unequal distribution of light source intensity, it was necessary to carry out black and white correction for hyperspectral images. The blackboard image was obtained by covering the camera lens with an opaque cover, and the whiteboard image was obtained using a Teflon white board. The correction publicity is as follows:

$$R=\frac{I_{R}-I_{B}}{I_{W}-I_{B}}$$

In the formula, *R* is the corrected image, which was used in the following processing; *I*_*R*_ is the original image; *I*_*W*_ is the whiteboard image; and *I*_*B*_ is the blackboard image.

**FIGURE 2:**
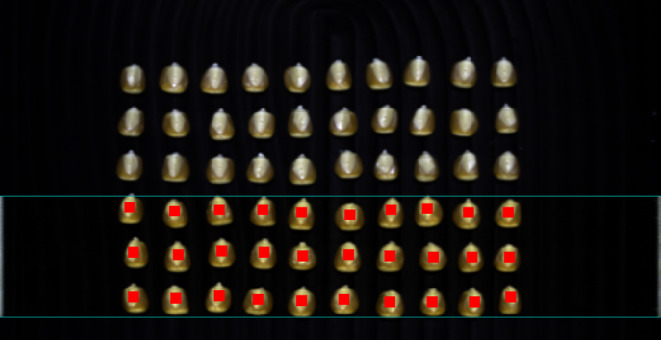
Schematic diagram of maize sample ROIs selection.

**FIGURE 3:**
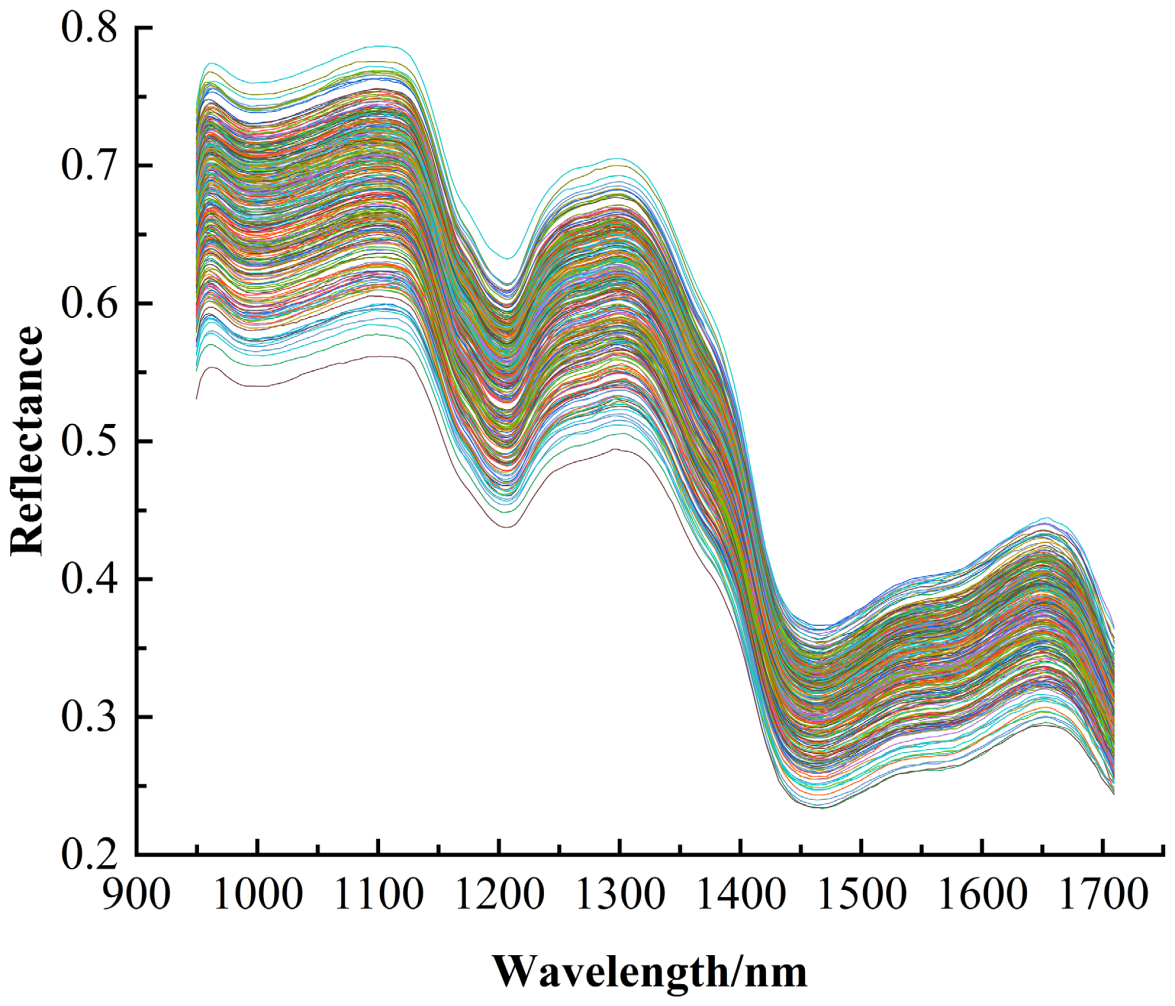
Average reflectance curve after interception.

After image correction, ENVI 5.3 was used to select ROIs of maize embryos with a size of 10 × 10 pixel, as shown in Figure 2. The average spectrum of all pixel points in the ROI region was extracted as the original spectral data of maize, and the original spectral information in the wavelength range of 935.61–1720.23 nm was obtained after processing. Since the original spectral data contained a large amount of random noise at both ends, the average spectral information in the range of 949.43–1709.49 nm was intercepted, and the average reflectance curve is shown in Figure 3.

### 2.4 Modeling methods and model evaluation criteria

CARS and SPA were used to reduce the original spectral dimension and improve the efficiency of model classification in the study. The CARS algorithm uses an adaptive reweighted sampling technique to select the wavelength points with a large absolute value of the regression coefficient in the PLS model, and interactive verification is used to select the subset with the lowest RMSECV value so as to find out the optimal combination of variables. The SPA algorithm is a feature selection method based on correlation that aims to select features that are highly related to the target variable from the original feature set. SVM was often used to solve nonlinear and high‐dimensional data model problems. It followed the principle of structural risk minimization and aimed to find the optimal hyperplane to maximize classification interval in high‐dimensional feature space, but classification accuracy was greatly affected by parameters (Feng et al., 2022). The penalty factor *c* and kernel parameter *g* were two important parameters when the RBF radial basis function was selected as the kernel function of SVM. GA is an optimization algorithm inspired by natural evolutionary theory with the characteristics of fast search speed, wide applicability, etc. The maximum number of evolutionary iterations was set to 200, the initial population was set to 20, and the search was terminated until the maximum number of iterations was reached when GA performed rapid parameter optimization of SVM. Finally, the *c* and *g* values were output to achieve parameter optimization (Liu et al., 2022). PSO also started from random solutions, setting the initial population number as 20, the maximum number of iterations as 200, the local search ability *C*_1_ as 1.5, the global search ability *C*_2_ as 1.7, and updating the speed and position of particles. PSO has the advantages of fast convergence and a simple algorithm to calculate the fitness value and determine whether it is a satisfactory solution (Cao et al., 2022).

After pretreatment, the spectral data in the range of 949.43–1709.49 nm were used to select characteristic wavelengths. The characteristic wavelengths and whole bands were taken as input variables, while the maize varieties were taken as output variables to establish SVM, GA‐SVM, and PSO‐SVM models. Identification accuracy, running time, and the number of bands were used as evaluation indexes to find the optimal model.

## 3 RESULTS AND ANALYSIS

### 3.1 Training set and test set division

There were a total of 300 samples in the experiment. Five types of maize samples were randomly divided into training sets and test sets at a ratio of 2:1, where each category training set and test set were 40 and 20, and the five categories training set and test set were 200 and 100, respectively.

### 3.2 Spectral data pretreatment

**FIGURE 4:**
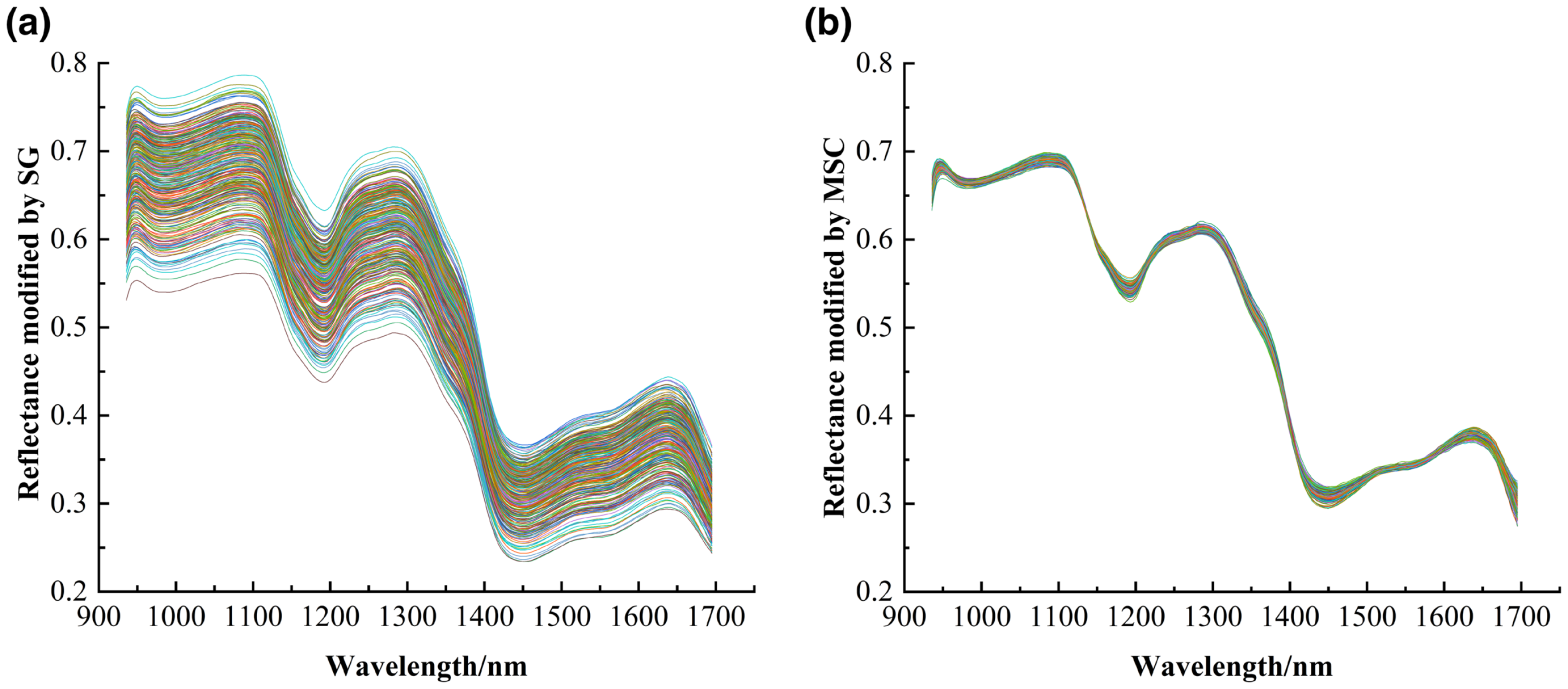
Spectral average reflectivity curve after pretreatment. (a) SG pretreatment; (b) MSC pretreatment.

There was a large amount of redundant information in the original full spectral band, which was interfered by irrelevant information such as noise, light scattering, and sample background. Direct application in modeling will reduce the overall classification accuracy of the model. Therefore, the raw spectral data needed to be pretreated to extract the effective information of the spectra and enhance the accuracy and stability of the model prediction. SG and MSC in the Unscrambler X 10.4 software were used to pretreat the spectral data of maize samples. The average spectral reflectance curve after pretreatment is shown in Figure 4.

### 3.3 Characteristic wavelength selection

#### 3.3.1 Competitive adaptive reweighted sampling (CARS)

**FIGURE 5:**
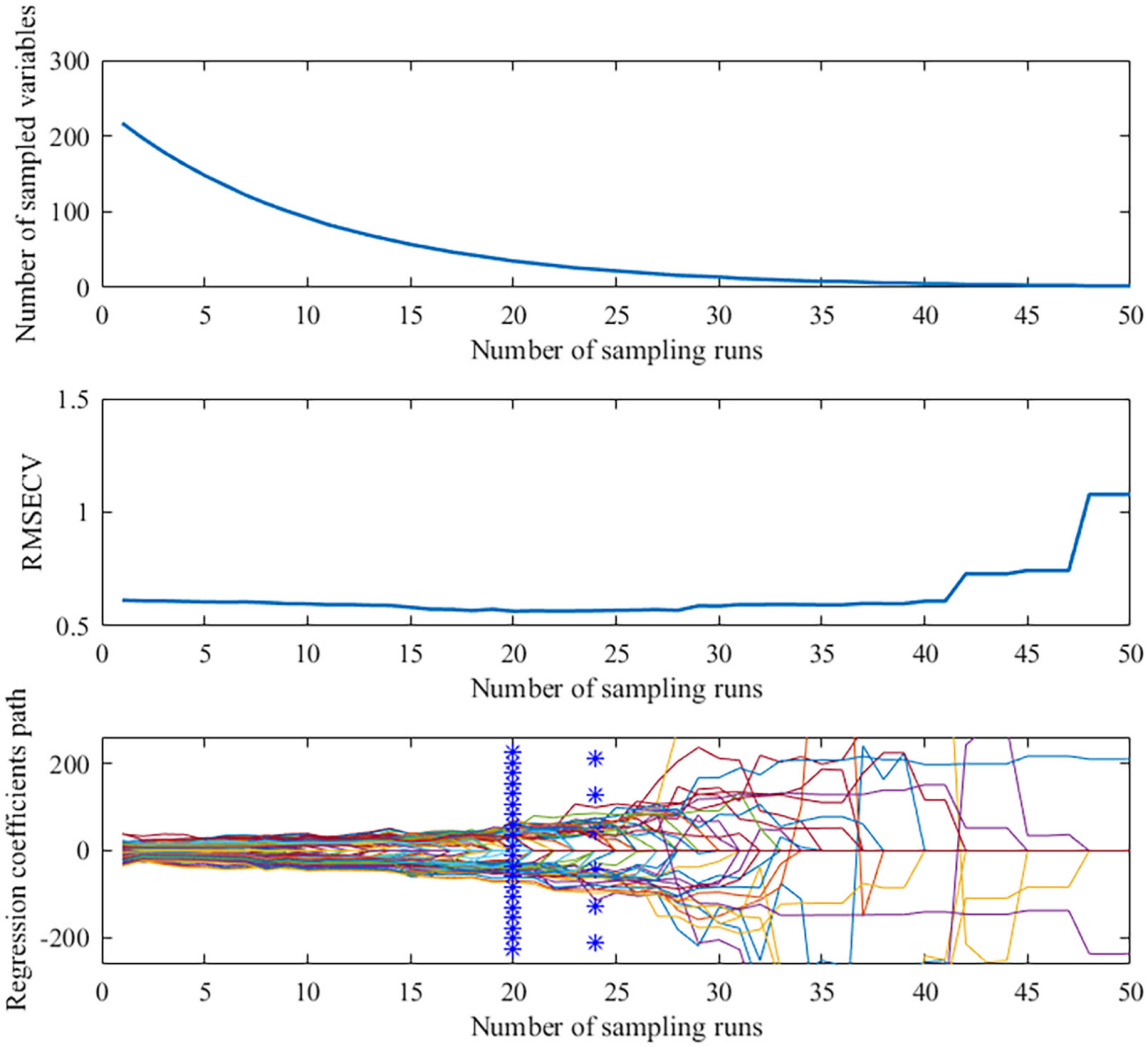
Characteristic wavelengths extracted by CARS after SG pretreatment. (a) Variation of the number of feature wavelength variables; (b) Variation of RMSECV; (c) Variation of regression coefficient.

**FIGURE 6:**
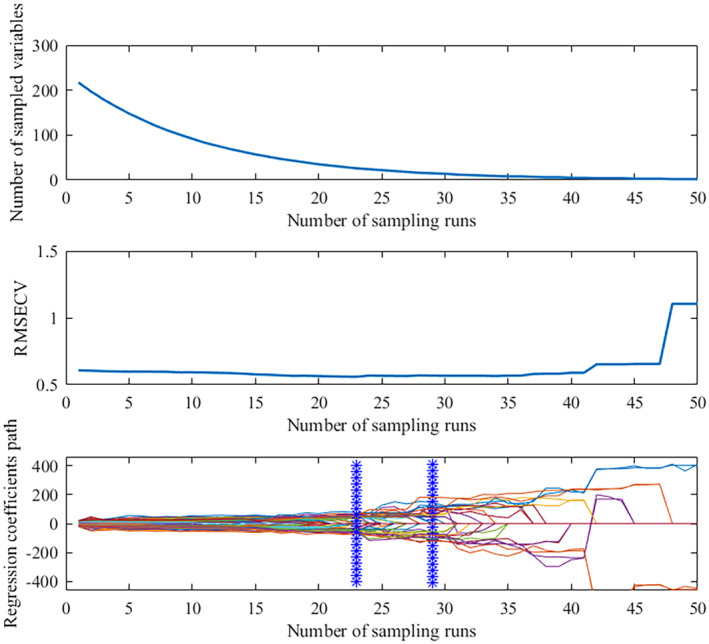
Characteristic wavelengths extracted by CARS after MSC pretreatment.

The number of Monte Carlo sampling times was set to 50, and the five‐fold cross‐validation method was used to extract the characteristic wavelengths of the spectral data by SG and MSC pretreatment, respectively. The results run in MATLAB 2016b are shown in Figures 5 and 6. Figure 5a showed that the number of selected wavelength variables decreased exponentially with the increase in sampling times. Before the 20th sampling, the number of selected characteristic wavelength variables decreased sharply while slowly after the 20th sampling. Figure 5b showed that the root mean square error of cross validation (RMSECV) rapidly decreased with the elimination of irrelevant information bands during the 1–20 sampling, which reached the minimum value at the 20th and increased gradually due to the elimination of effective information related to the identification of maize varieties. Figure 5c shows the minimum RMSECV value at the 20th and 24th sampling times. Finally, 35 key wavelength variables were selected based on the principle of selecting the optimal variable combination with the lowest RMSECV. The RMSECV value shown in Figure 6 was the smallest at the 23rd and 29th samplings when selecting the spectral data after MSC pretreatment, and 26 characteristic wavelength variables were determined and selected.

#### 3.3.2 Successive projection algorithm (SPA)

**FIGURE 7:**
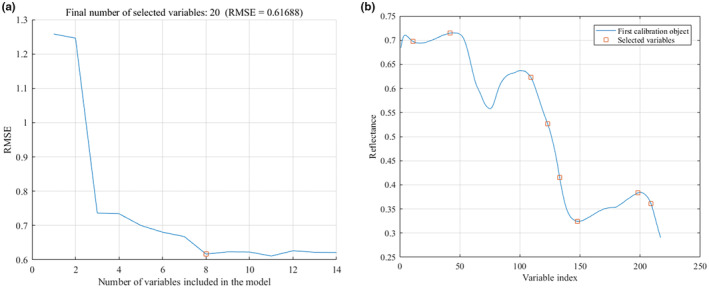
Characteristic wavelengths extracted by SPA after SG pretreatment. (a) Number of variables; (b) Variables distribution.

**FIGURE 8:**
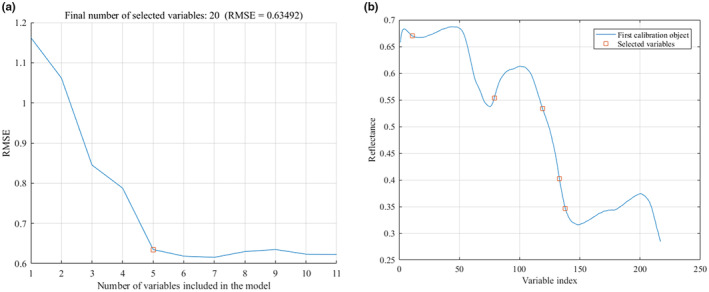
Characteristic wavelengths extracted by SPA after MSC pretreatment. (a) Number of variables; (b) Variables distribution.

The maximum number of optimal wavelengths was set to 20 in the SPA optimization process. With the increase of the number of characteristic wavelengths, the root mean square error (RMSE) value decreased first and then became gentle, indicating that there were no redundant wavelengths to be removed at this time. Eight characteristic wavelength variables were optimized for the spectral data after SG pretreatment, and the minimum value of RMSE was 0.61688, as shown in Figure 7. For the MSC pretreatment data, five characteristic wavelengths were selected, and the minimum value of RMSE was 0.63492, as shown in Figure 8.

#### 3.3.3 Combination and secondary screening of characteristic wavelengths (CARS + SPA, CARS‐SPA)

The objective of this study was to solve the problem of missing information about some key variables and the decreasing accuracy of model predictions caused by the single screening of CARS and SPA. The characteristic wavelengths were extracted by the combination of CARS and SPA, which took the union of characteristic wavelengths. There was still redundant information between bands extracted by single screening in some cases. Therefore, the characteristic variables extracted by CARS were screened again by SPA, which was CARS‐SPA secondary screening, in order to remove redundancy and collinearity information between bands and achieve data dimension reduction. The results of different extraction methods are shown in Table 1.

**TABLE 1:** The number of wavelength extracted.

Pretreatment methods	CARS	SPA	CARS + SPA	CARS‐SPA
SG	35	8	38	10
MSC	26	5	29	8

**FIGURE 9:**
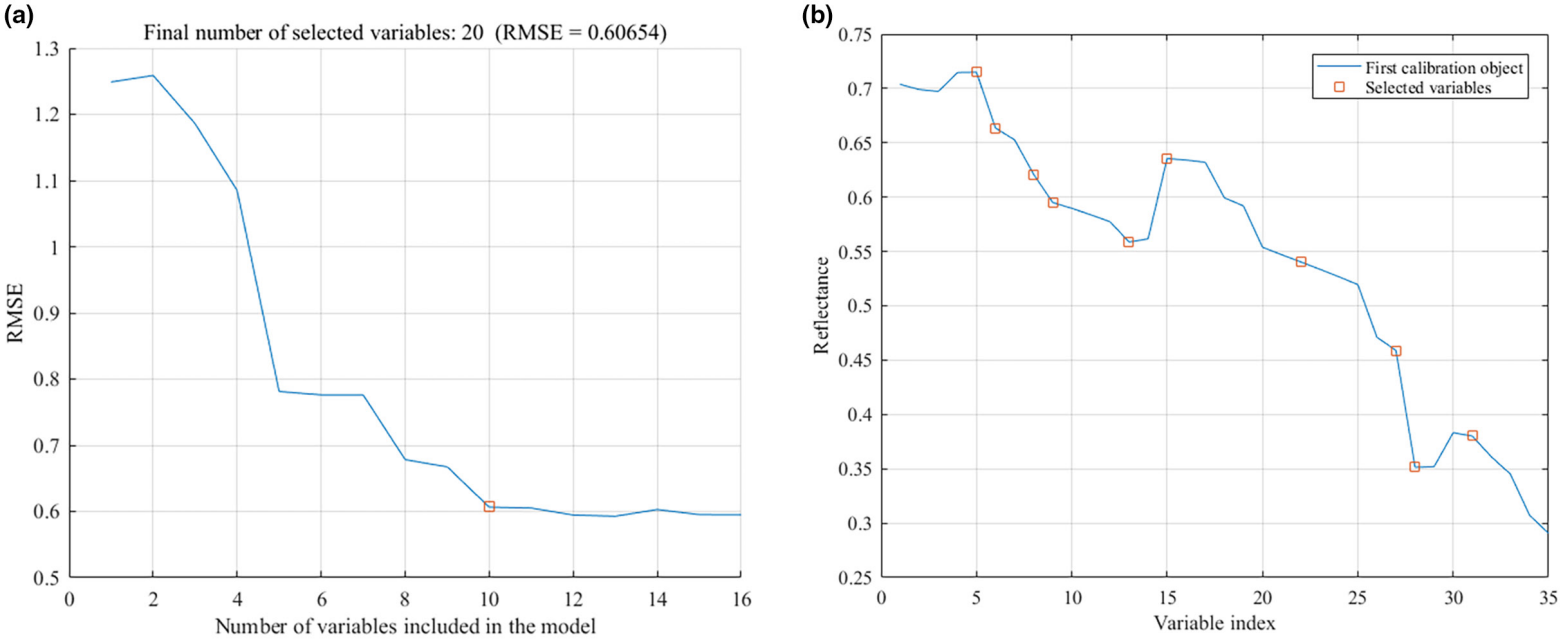
Characteristic wavelengths extracted by CARS‐SPA after SG pretreatment. (a) Number of variables; (b) Variables distribution.

**FIGURE 10:**
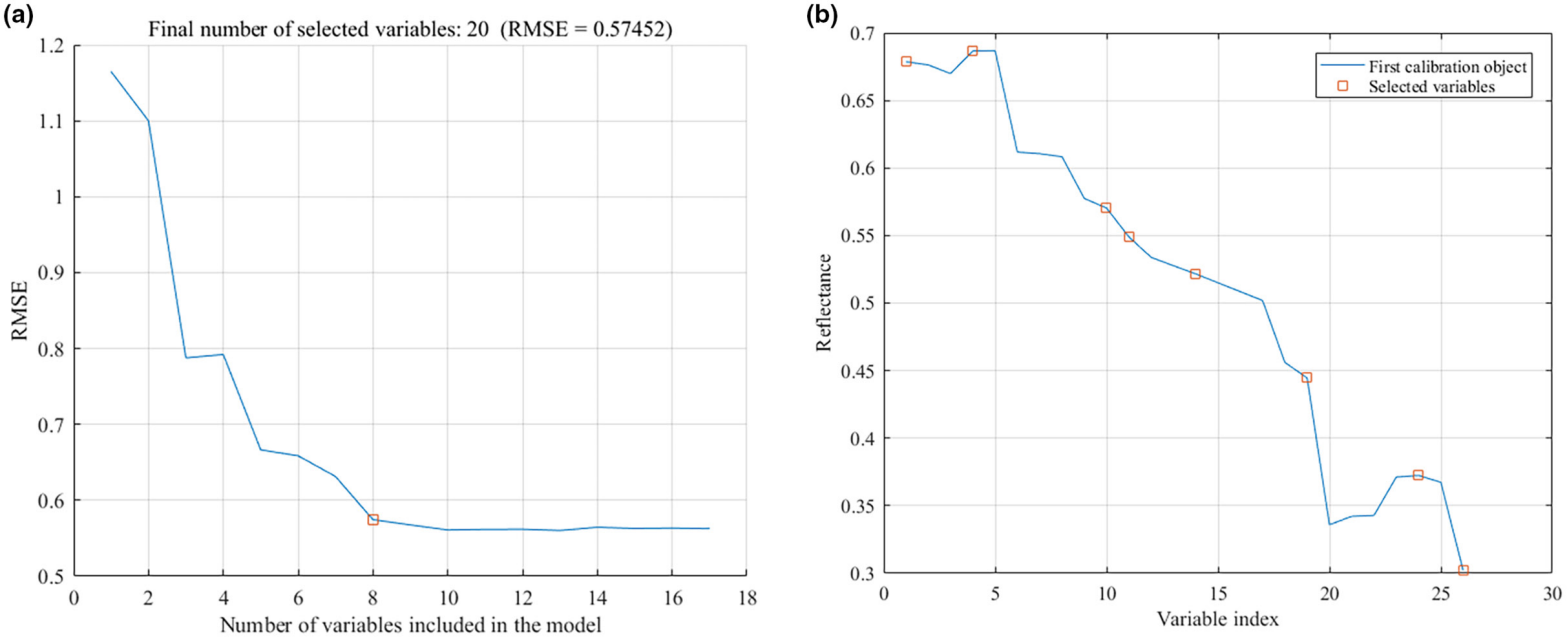
Characteristic wavelengths extracted by CARS‐SPA after MSC pretreatment. (a) Number of variables; (b) Variables distribution.

Using CARS‐SPA to extract characteristic variables from the SG and MSC pretreatment data. For the SG pretreatment data, the minimum value of RMSE was 0.60654, the number of feature wavelengths was 10, and the extraction results are shown in Figure 9. For the MSC pretreatment data, the minimum value of RMSE was 0.57452, the number of feature wavelengths was 8, and the extraction results are shown in Figure 10.

### 3.4 Model building and analysis

Based on the three classification models of SVM, GA‐SVM, and PSO‐SVM, full bands and characteristic wavelengths after single screening of CARS and SPA, combined screening of CARS + SPA, and secondary screening of CARS‐SPA were taken as input variables, while maize variety categories were taken as output variables. The performance of the model was evaluated by classification accuracy. The results are shown in Tables 2, 3, 4.

**TABLE 2:** Classification accuracy of SVM model.

Models	SG		MSC	
	Training set/%	Test set/%	Training set/%	Test set/%
FB‐SVM	52	48	55	53
CARS‐SVM	41	43	55	53
SPA‐SVM	38.5	41	65.5	59
CARS+SPA‐SVM	41	45	62	61
CARS‐SPA‐SVM	39	41	62	61

**TABLE 3:** Classification accuracy of GA‐SVM model.

Models	SG		MSC	
	Training set/%	Test set/%	Training set/%	Test set/%
**FB‐GA‐SVM**	86	75	99	**95**
CARS‐GA‐SVM	93	73	99	87
SPA‐GA‐SVM	87.5	76	90.5	80
**CARS+SPA‐GA‐SVM**	93	74	97	**90**
**CARS‐SPA‐GA‐SVM**	90.5	74	91	**93**

The bolded values in Table 3 represent better models which need to be selected twice.

**TABLE 4:** Classification accuracy of PSO‐SVM model.

Models	SG		MSC	
	Training set/%	Test set/%	Training set/%	Test set/%
FB‐PSO‐SVM	78	72	98.5	92
CARS‐PSO‐SVM	94	74	100	85
SPA‐PSO‐SVM	90.5	73	90.5	80
CARS+SPA‐PSO‐SVM	94	74	98	88
CARS‐SPA‐PSO‐SVM	90	74	98	87

The classification results of maize varieties by different modeling methods are shown in tables. It can be seen that the prediction accuracy of the model is directly affected by the spectral pretreatment. Besides, the test accuracy after MSC pretreatment is 4%–20% higher than that after SG pretreatment under the same model, indicating that MSC can reduce the influence of baseline shift and is more suitable for the establishment of a maize variety identification model. Therefore, the subsequent analysis is based on the spectral data after MSC pretreatment. The SVM model generated parameter values randomly, resulting in its extremely low classification accuracy. In order to test the performance of the optimized model, GA‐SVM and PSO‐SVM were selected for comparison. The results show that under the same feature wavelength extraction methods, the classification accuracy of the GA‐SVM model is higher than that of PSO‐SVM, and the classification accuracy of both optimization models is significantly increased by about 30%–40% compared with that of SVM. Among them, MSC‐FB‐GA‐SVM, MSC‐(CARS + SPA)‐GA‐SVM, and MSC‐(CARS‐SPA)‐GA‐SVM all achieved discriminative accuracy of 90% and above, which were 95%, 90%, and 93%. In order to find an optimal model with high classification accuracy and a short running time, the above three models were preferentially selected again, and the model performance comparison is shown in Table 5.

**TABLE 5:** Secondary comparison of model performance.

Pretreatment methods	Modeling method	Accuracy/%	Number of misjudgments	Time/s	Number of variables
MSC	FB‐GA‐SVM	95	5	133.20	217
	CARS+SPA‐GA‐SVM	90	10	35.38	29
	**CARS‐SPA‐GA‐SVM**	**93**	**7**	**24.45**	**8**

The bolded values in Table 5 represent the best results of the best model.

By comprehensive comparison, it could be observed that the test set discrimination accuracy of MSC‐FB‐GA‐SVM is 95% at the highest. However, full‐band spectral data processing requires a large amount of calculation and a long running time of 133.20s, which is not conducive to the establishment of classification models. The discriminative accuracy of the MSC‐(CARS‐SPA)‐GA‐SVM model is 93%, with the shortest running time of 24.45 s. Compared with the number of variables modeled, the characteristic wavelength used is the least of 8, reduced by 96.31% compared with full bands, indicating that the characteristic wavelength of CARS‐SPA secondary screening can represent the main spectral information of samples and meet the requirements of fast and accurate detection. The 8 characteristic wavelengths were 949.43, 959.81, 980.58, 984.04, 994.43, 1011.77, 1029.12, and 1036.06 nm. CARS + SPA‐GA‐SVM is the second. Therefore, the model MSC‐(CARS‐SPA)‐GA‐SVM showed the best performance. The confusion matrix of the best classification model is shown in Table 6. There were 20 maize varieties in each category, with a total of 100 grains in the test set. A total of 93 were predicted accurately among them, with an accuracy of 93%. All the predictions in categories 1, 3, and 4 were correct, while categories 2 and 5 had 2 and 5 prediction errors, respectively.

**TABLE 6:** Confusion matrix of MSC‐(CARS‐SPA)‐GA‐SVM.

Confusion matrix	Predict					Total
	Class 1	Class 2	Class 3	Class 4	Class 5	
Ground truth						
Class 1	20	0	0	0	0	20
Class 2	1	18	1	0	0	20
Class 3	0	0	20	0	0	20
Class 4	0	0	0	20	0	20
Class 5	0	1	0	4	15	20
Total	21	19	21	24	15	

## 4 CONCLUSION

In this study, spectral data of maize seeds were collected by the hyperspectral imaging system. The classification models were established after pretreatment and characteristic wavelength extraction. The method of spectral pretreatment had a direct impact on the accuracy of model identification. MSC pretreatment can better eliminate redundant information unrelated to the properties of the sample in the spectral data, optimize the spectral data, and effectively improve the accuracy of the model. Therefore, the data after MSC preprocessing was selected for the following research. The number of characteristic wavelengths extracted by CARS, SPA, CARS + SPA, and CARS‐SPA was reduced to 88.02%, 97.70%, 86.64%, and 96.31% compared with full bands pretreated by MSC, which simplified models. The SVM model generated parameters c and g randomly, resulting in low accuracy. GA and PSO algorithms were used to iteratively select the optimal parameter combination. It was found that the optimization effect of GA was better than that of PSO and more suitable for the identification of maize varieties. Three models with more than 90% prediction accuracy were optimized twice. The discrimination accuracy of the test set was 93% in the (CARS‐SPA)‐GA‐SVM model, while it was 90% in the (CARS + SPA)‐GA‐SVM, and 95% in the FB‐GA‐SVM. However, the identification time of (CARS‐SPA)‐GA‐SVM was only 24.45 s, which was 108.75 s less than that of FB‐GA‐SVM. Therefore, the (CARS‐SPA)‐GA‐SVM was the best model for identifying the varieties of maize and provided ideas and references for the research related to variety identification.

## AUTHOR CONTRIBUTIONS

**Fu Zhang:** Conceptualization (equal); formal analysis (equal); methodology (equal); project administration (lead); writing – original draft (equal); writing – review and editing (equal). **Mengyao Wang:** Conceptualization (equal); data curation (equal); formal analysis (equal); investigation (equal); writing – original draft (equal); writing – review and editing (equal). **Fangyuan Zhang:** Conceptualization (equal); formal analysis (equal); investigation (equal); visualization (equal); writing – original draft (equal); writing – review and editing (equal). **Ying Xiong:** Resources (equal); validation (equal); writing – review and editing (equal). **Xinyue Wang:** Data curation (equal); resources (equal); visualization (equal). **Qingfeng Lv:** Conceptualization (equal); resources (equal). **Yakun Zhang:** Conceptualization (equal); methodology (equal). **Sanling Fu:** Conceptualization (equal); funding acquisition (equal); project administration (equal); supervision (equal).

## Data Availability

The data is not shared.
